# Ergotamine and triptans induced medication-overuse headache: a real-world population-based comparative study from the Northern Thai headache registry

**DOI:** 10.1186/s10194-025-02161-6

**Published:** 2025-10-02

**Authors:** Surat Tanprawate, Kitti Thiankhaw, Watthikorn Chusilthong, Vipanee Muangchean, Sirinada Ma-imjai, Noratham Chalapati, Kanokkarn Teekaput, Chutithep Teekaput

**Affiliations:** 1 Division of Neurology, Department of Internal Medicine, Faculty of Medicine, Chiang Mai University, Chiang Mai, Thailand; 2 The Northern Neuroscience Center, Faculty of Medicine, Chiang Mai University, Chiang Mai, Thailand; 3 Faculty of Medicine, Chiang Mai University, Chiang Mai, Thailand; 4 Chiang Mai Municipal Hospital, Chiang Mai, Thailand; 5 Department of Neurology, Mayo Clinic, Rochester, MN USA

**Keywords:** Migraine, Medication-overuse headache, Outcomes, Withdrawal symptoms

## Abstract

**Background:**

Medication-overuse headache (MOH) remains a leading cause of chronic daily headache globally. Although triptans are the predominant implicated in high-income countries, ergotamine continues to be widely used in many low- and middle-income settings. Comparative data on ergotamine- versus triptan-induced MOH remain limited.

**Methods:**

We analyzed prospective data from the Northern Thai Headache Registry, enrolling patients with ergotamine-MOH, triptan-MOH, or dual-MOH. Demographic and clinical characteristics, treatment outcomes, recurrence rates, and withdrawal symptoms were evaluated over 12 months. Outcomes included changes in Headache Impact Test (HIT-6) scores, recurrence-free survival, and adverse events during withdrawal.

**Results:**

A total of 117 MOH patients were included: 61 (52.1%) with ergotamine-MOH, 44 (37.6%) with triptan-MOH, and 12 (10.3%) with dual-MOH. Patients with ergotamine-MOH experienced significantly more withdrawal symptoms compared to those with triptan-MOH (*P* < 0.01). In contrast, recurrence rates were highest among dual-MOH patients (*P* < 0.01). Improvement in HIT-6 scores was observed across all groups, though ergotamine-MOH patients showed slower recovery trajectories. Kaplan–Meier analysis demonstrated a higher risk of recurrence in dual-MOH compared to single-agent MOH.

**Conclusions:**

Ergotamine-induced MOH is linked to more severe withdrawal symptoms, whereas dual-MOH carries the greatest risk of recurrence. These findings highlight the importance of tailored withdrawal strategies and close monitoring, particularly in resource-limited settings where ergotamine remains widely available. They also support policy initiatives aimed at restricting over-the-counter ergotamine and expanding access to safer acute treatment options.

**Supplementary Information:**

The online version contains supplementary material available at 10.1186/s10194-025-02161-6.

## Introduction

Medication-overuse headache (MOH) is a significant clinical concern characterized by the chronic and excessive use of abortive medications, leading to a worsening headache disorder and substantial impairment in daily functioning and quality of life [1]. The prevalence of MOH varies across studies, ranging from 1% to 2% in the global population to 50% in a tertiary care hospital [2, 3]. Patients with MOH frequently encounter considerable disability, diminished productivity, and reduced quality of life, emphasizing the need for effective management strategies [4, 5]. Standard treatments typically involve medication withdrawal combined with preventive therapeutic approaches aimed at reducing headache frequency, severity, and recurrence [6–9]. A notable challenge in MOH treatment is managing withdrawal symptoms, which differ by drug class and may include severe headaches, gastrointestinal distress, sleep disturbances, anxiety, and agitation [3, 10].

Despite global shifts toward triptans as the preferred first-line abortive therapy, ergotamine remains widely used in limited resource countries, including Thailand due to its accessibility and affordability [11]. Its over-the-counter availability increases the risk of misuse, contributing to a higher incidence of ergotamine-associated MOH in the region [12, 13]. Thailand offers a unique case study in this regard. The continued accessibility of ergotamine, often in fixed-dose combinations with caffeine, creates a therapeutic landscape that is rarely seen in Western settings, where ergotamine use has largely disappeared. This provides a valuable opportunity to systematically compare ergotamine- and triptan-induced MOH in real-world clinical practice, with implications both locally and for other regions where ergotamine remains available. These two drug classes also represent the most clinically relevant contrasts: ergotamine, with its broad receptor profile and caffeine combinations, is associated with more intense withdrawal symptoms and higher relapse risk, whereas triptans, as more selective 5-HT1B/1D agonists, are generally considered safer but with less well-defined long-term outcomes. Such differences highlight key questions regarding withdrawal severity, functional recovery, recurrence risk, and policy implications in settings where ergotamine remains widely accessible.

Given these contextual factors, our study aimed to evaluate the clinical characteristics of withdrawal symptoms associated with ergotamine, triptan, and dual (ergotamine combined with triptan) medication overuse. Additionally, we sought to assess treatment patterns, patient demographics and clinical characteristics, and functional outcomes, including changes in headache frequency, the Headache Impact Test-6 (HIT-6) scores, and overall headache-related disability following detoxification therapy. By elucidating these characteristics, our findings aim to inform better clinical management strategies and improve outcomes for patients with MOH.

## Methods

### Study design and population

In this single-center, retrospective study, data were retrieved from the Chiang Mai University Hospital Headache Registry from 2019 to 2024. This registry prospectively collected information on consecutive patients diagnosed with various types of headache syndromes. Only patients diagnosed with MOH specifically attributed to migraine-specific abortive medications were included in this study. Eligible participants were categorized into three distinct groups based on the type of medication overused: ergotamine-MOH (patients who exclusively overused ergotamine), triptan-MOH (patients who exclusively overused triptans), and dual-MOH (patients who overused ergotamine and triptans). In Thailand, ergotamine is available only as a fixed-dose combination with caffeine; therefore, all references to ‘ergotamine’ in this study indicate the use of ergotamine–caffeine. The study received approval from the Institutional Review Board of the Faculty of Medicine, Chiang Mai University (Study code: MED-2567-0195). All data from this study were fully anonymized, and confidentiality was maintained throughout data handling. Since the study data were collected as part of routine clinical care, the requirement for individual patient consent was waived by the Research Ethics Committee.

### Data collection and protocol

All patients aged 18 years or older who met the diagnostic criteria for MOH according to the International Classification of Headache Disorders, 3rd edition (ICHD-3), were enrolled in the study [14]. Board-certified neurologists subsequently reviewed clinical characteristics, including age, sex, migraine history, migraine severity, underlying comorbidities, and both abortive and preventive medications previously or currently used. In accordance with institutional and national guidelines, patients with MOH were evaluated to determine whether they would undergo treatment via outpatient or inpatient protocols [15]. This decision-making process involved a collaborative evaluation by certified neurologists and a comprehensive care team. Patients underwent standardized institutional detoxification protocols, with inpatient management prioritized for those with severe symptoms. The protocol included abrupt discontinuation of overused medications (including ergotamine, triptans, NSAIDs, or opioids), steroid administration, intravenous hydration, and preventive therapy initiation. Follow-up visits occurred regularly at intervals ranging from one to three months. During follow-up, patients were asked to record headache diaries in either electronic or paper-based formats. These diaries systematically captured data on headache frequency, severity, medication usage, and associated symptoms. All participants were monitored for a minimum duration of 12 months post-detoxification to evaluate the effectiveness of the detoxification protocols and to identify cases of recurrent MOH.

### HIT-6 and satisfaction score

Patients’ headache-related disability and the impact on daily activities were evaluated using the HIT-6 [16, 17]. The HIT-6 measures the impact of headaches on daily life through six questions addressing pain severity, social functioning, role functioning, cognitive functioning, psychological distress, and vitality. Scores range from 36 to 78, with higher scores indicating greater headache-related disability: ≤ 49 = little impact, 50–55 = some impact, 56–59 = substantial impact, and ≥ 60 = severe impact [16]. In the study, the HIT-6 was assessed at baseline, 3 months, and 6 months post-detoxification. The satisfaction score was assessed at the 3-month follow-up using a visual analog scale (VAS), ranging from 0 to 10, where 0 indicates the lowest level of satisfaction and 10 denotes the highest level of satisfaction.

### Study outcomes

The primary outcome was the comparison of withdrawal symptom characteristics among patients with ergotamine-MOH, triptan-MOH, and dual-MOH. Secondary outcomes included clinical parameters such as HIT-6, headache frequency, and satisfaction score, as well as treatment profiles and MOH recurrence. HIT-6 scores and headache frequency were evaluated at three distinct time points: baseline, 1-month follow-up, and 3-month follow-up. Changes in HIT-6 scores and headache frequency from baseline to follow-up visits were analyzed to determine improvements attributable to detoxification treatment. Patient satisfaction scores were obtained at the 3-month follow-up using a VAS ranging from 0 (least satisfied) to 10 (most satisfied). Patients were monitored for at least 12 months post-treatment to identify any recurrence of MOH. Additional data, including symptoms, use of abortive medication, and headache frequency, were systematically collected from patient migraine diaries throughout the follow-up period.

### Statistical analysis

Clinical data were presented as numbers and percentages for categorical variables, and as mean ± standard deviation (SD) or median with interquartile range (IQR) for continuous variables, as appropriate. Group comparisons for categorical data were performed using the Pearson’s χ² test or Fisher’s exact test, as applicable. For continuous variables, comparisons between two groups were conducted using Student’s t-test or Mann–Whitney U test, depending on normality, while comparisons across three groups were assessed using one-way ANOVA or the Kruskal–Wallis test as appropriate. When the Kruskal–Wallis test indicated a statistically significant overall difference, Dunn’s post hoc test was used for pairwise comparisons, and Bonferroni correction was applied to adjust for multiple testing (significance threshold set at *P*-value < 0.02). To assess factors associated with withdrawal symptoms, univariable logistic regression was first performed to calculate crude odds ratios (OR) with 95% confidence intervals (CI). Multivariable logistic regression was then used to adjust for potential confounders, including age, acetaminophen, non-steroidal anti-inflammatory drugs (NSAIDs), opioid use, HIT-6 score, baseline monthly headache days, and prior preventive medication use [18, 19]. To assess treatment response over time within each MOH group, the Wilcoxon signed-rank test was used to compare baseline vs. 30-day and baseline vs. 90-day values for HIT-6 scores and monthly headache days. The association between MOH group and time to relapse was evaluated using Cox proportional hazards models, after confirming the proportional hazards assumption with Schoenfeld’s residuals. Kaplan–Meier survival analysis was used to illustrate MOH-free survival probability over time across treatment groups. All statistical tests were two-sided, and a *P*-value < 0.05 was considered statistically significant unless adjusted otherwise. Variables with more than 50% missing data or lacking follow-up outcomes were excluded from the analysis. For the remaining variables, complete-case analysis was applied, as the proportion of missing data was low and assumed to be missing at random. Analyses were conducted using licensed Stata software version 16.1 (StataCorp, College Station, TX, 2019) and GraphPad Prism version 10.0 (GraphPad Software, USA).

## Results

### Baseline characteristics of ergotamine, triptans and dual MOH

**Fig. 1:**
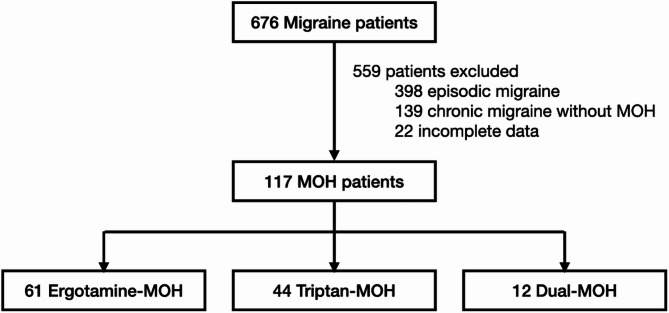
Flow chart of the study. MOH, medication-overuse headache

Among 676 migraine patients, 559 were excluded: 398 diagnosed with episodic migraine, 139 diagnosed with chronic migraine without evidence of MOH, and 22 due to incomplete data. The remaining 117 patients were classified as having MOH and assigned to one of three treatment groups: ergotamine-MOH (*n* = 61), triptan-MOH (*n* = 44), or dual-MOH (*n* = 12) (Fig. 1). The mean age of participants was 39.0 ± 14.1 years, with a predominance of female patients (80.3%). Across the three MOH groups, no significant differences were observed in key baseline characteristics, common migraine triggers, baseline headache frequency, concomitant use of migraine non-specific abortive treatments, and migraine comorbidities (Table 1). The HIT-6 score was statistically higher in the dual-MOH group compared to the other groups (56.0, 57.0 and 66.0 points for ergotamine-, triptan- and dual-MOH, respectively, *P*-value = 0.02), reflecting a greater headache burden. Importantly, this between-group difference remained statistically significant after post hoc analysis and Bonferroni adjustment, particularly between ergotamine and triptan groups (*P*-value = 0.02). Among the total cohort, 26.5% had migraine with aura, with visual aura (93.6%) being the most frequently reported, followed by sensory aura (25.8%). Regarding preventive therapy, 56.4% of patients had been prescribed at least one preventive medication. Among these, tricyclic antidepressants (TCAs) were the most frequently used, at 63.6%, followed by topiramate at 50.0%, and selective serotonin reuptake inhibitors (SSRIs) and serotonin-norepinephrine reuptake inhibitors (SNRIs) at 39.4%.

**Table 1:** Clinical characteristics among patients with ergotamine-MOH, triptan-MOH and dual-MOH

Parameters	Total (*n* = 117)	Ergotamine (*n* = 61)	Triptans (*n* = 44)	Dual (*n* = 12)	*P*-value*			
					Ergotamine vs. triptans	Ergotamine vs. Dual	Triptans vs. Dual	Overall
Female – n (%)	94 (80.3)	49 (80.3)	34 (77.3)	11 (91.7)	0.81	0.68	0.42	0.54
Age, years - mean ± SD	39.0 ± 14.1	40.2 ± 11.6	37.8 ± 9.8	37.6 ± 14.1	0.21	0.46	0.64	0.50
Weight, kg – median (IQR)	65.0 (56.0, 78.0)	61.0 (56.0, 74.0)	66.0 (56.5, 80.5)	67.0 (58.0, 79.0)	0.17	0.37	0.94	0.33
Age of diagnosis, years – median (IQR)	33.0 (23.0, 40.0)	33.0 (23.0, 43.0)	33.5 (23.5, 40.0)	32.0 (23.0, 34.0)	0.95	0.33	0.28	0.33
First migraine diagnosis at this visit– n (%)	52 (44.4)	27 (44.3)	18 (40.9)	7 (58.3)	0.84	0.53	0.34	0.56
Triggering factors – n (%)								
Stress	79 (67.5)	42 (68.9)	28 (63.6)	9 (75.0)	0.68	1.00	0.73	0.72
Light	39 (33.3)	22 (36.1)	15 (34.1)	2 (16.7)	1.00	0.32	0.31	0.42
Loud noise	22 (18.8)	14 (23.0)	6 (13.6)	2 (16.7)	0.32	1.00	1.00	0.47
Sleep disturbance	59 (50.4)	31 (50.8)	21 (47.7)	7 (58.3)	0.84	0.76	0.75	0.81
Odor	40 (34.2)	25 (41.0)	10 (22.7)	5 (41.7)	0.06	1.00	0.27	0.13
Heat	51 (43.6)	28 (45.9)	18 (40.9)	5 (41.7)	0.69	1.00	1.00	0.87
Migraine days per month, days – median (IQR)	17.0 (15.0, 19.0)	17 (15.0, 19.0)	17.0 (15.0, 18.5)	16.0 (15.0, 19.0)	0.92	0.98	0.90	0.99
HIT-6, points - median (IQR)	56.0 (53.0, 65.0)	56.0 (50.0, 58.0)	57.0 (55.0, 67.0)	66.0 (54.0, 70.0)	0.02^†^	0.05	0.41	0.02^†^
Pain score, points - median (IQR)	8.0 (7.0, 8.0)	8.0 (7.0, 8.0)	7.0 (7.0, 8.0)	7.5 (6.5, 8.0)	0.20	0.39	0.97	0.38
Migraine with aura – n (%)	31 (26.5)	18 (29.5)	9 (20.5)	4 (33.3)	0.37	0.75	0.44	0.89
Aura – n (%) (*n* = 31)								
Visual	29 (93.6)	16 (88.9)	9 (100.0)	4 (100.0)	0.54	1.00	1.00	0.46
Speech	2 (6.5)	2 (11.1)	0/9 (0.0)	0 (0.0)	0.54	1.00	1.00	0.46
Sensory	8 (25.8)	4 (22.2)	3 (33.3)	1 (25.0)	0.65	1.00	1.00	0.82
Motor	2 (6.5)	1 (5.6)	1 (11.1)	0 (0.0)	1.00	1.00	1.00	0.73
Vestibular	2 (6.5)	2 (11.1)	0 (0.0)	0 (0.0)	0.54	1.00	1.00	0.46
Retinal	2 (6.5)	1 (5.6)	1 (11.1)	0 (0.0)	1.00	1.00	1.00	0.73
Concomitant abortive medication								
Acetaminophen – n (%)	101 (86.3)	50 (82.0)	41 (93.2)	10 (83.3)	0.15	1.00	0.29	0.24
Acetaminophen per month, days - median (IQR)	13.0 (8.0, 16.0)	12.0 (8.0, 16.0)	13.0 (10.0, 15.0)	16.0 (6.0, 20.0)	0.83	0.18	0.17	0.36
NSAIDs – n (%)	111 (95.7)	56 (93.3)	43 (97.7)	12 (100.0)	0.39	1.00	1.00	0.41
NSAIDs per month, days - median (IQR)	15.0 (12.0, 16.0)	15.0 (12.0, 17.0)	15.0 (12.0, 16.0)	15.0 (13.0, 15.5)	0.32	0.50	0.95	0.56
Opioid – n (%)	21 (18.0)	15 (24.6)	3 (6.8)	3 (25.0)	0.02^†^	1.00	0.11	0.05
Opioid day per month, days - median (IQR)	9.0 (8.0, 10.0)	9.0 (8.0, 10.0)	7.0 (6.0, 8.0)	8.0 (8.0, 9.0)	0.004^†^	0.10	0.14	0.02^†^
Prior preventive medication – n (%)								
Prior preventive medication	66 (56.4)	35 (57.4)	26 (59.1)	5 (41.7)	1.00	0.36	0.34	0.55
Topiramate	33/66 (50.0)	21/35 (60.0)	10/26 (38.5)	2/5 (40.0)	0.12	0.63	1.00	0.23
Valproate	25/66 (37.9)	11/35 (31.4)	12/26 (46.2)	2/5 (40.0)	0.29	1.00	1.00	0.50
Beta-blockers	13/66 (19.7)	6/35 (17.1)	7/26 (26.9)	0/5 (0.0)	0.53	1.00	0.56	0.33
Flunarizine	14/66 (21.2)	6/35 (17.1)	6/26 (23.1)	2/5 (40.0)	0.75	0.26	0.58	0.48
TCAs	42/66 (63.6)	23/35 (65.7)	16/26 (61.5)	3/5 (60.0)	0.79	1.00	1.00	0.93
SSRI/SNRIs	26/66 (39.4)	13/35 (37.1)	12/26 (46.2)	1/5 (20.0)	0.60	0.64	0.37	0.51
History of MOH	25 (31.4)	13 (21.3)	11 (25.0)	1 (8.3)	0.81	0.44	0.43	0.46
Comorbidities – n (%)								
Anxiety	21 (18.0)	10 (16.4)	9 (20.5)	2 (16.7)	0.62	1.00	1.00	0.86
Depression	41 (35.0)	20 (32.8)	16 (36.4)	5 (41.7)	0.84	0.74	0.75	0.82
Myofascial pain syndrome	21 (18.0)	11 (18.0)	8 (18.2)	2 (16.7)	1.00	1.00	1.00	0.99

*Abbreviations: IQR* Interquartile range, *MOH* Medication-overuse headache, *NSAIDs* Non-steroidal anti-inflammatory drugs, *SD* Standard deviation, *SNRI* Serotonin-norepinephrine reuptake inhibitor, *SSRI* Selective serotonin reuptake inhibitor, *TCA* Tricyclic antidepressant

*Pairwise comparison *P*-values were Bonferroni-adjusted, with a significance threshold of *P* < 0.02. For overall comparisons, a *P*-value < 0.05 was considered statistically significant

^†^Statistically significant

### Withdrawal symptoms

Table 2 shows the distribution of withdrawal symptoms across the three MOH groups. Overall, withdrawal symptoms were reported in 87.0% of patients in the ergotamine-MOH group, 61.4% in the triptan-MOH group, and 83.3% in the dual-MOH group (overall *P*-value = 0.01). The ergotamine group had a significantly higher proportion of withdrawal symptoms compared to the triptan group (*P*-value = 0.01). Nausea and vomiting were significantly more frequent in both the dual-MOH (50.0%) and ergotamine-MOH (36.1%) groups compared to the triptan-MOH group (0.0%, both *P*-value < 0.001). Similarly, loss of appetite was more common in the ergotamine-MOH group (19.7%) than in the triptan-MOH group (2.3%, *P*-value = 0.008). Rebound headache was more frequent in the ergotamine group (57.4%) compared to the triptan group (25.0%, *P*-value = 0.001), but not significantly different from the dual group (50.0%, *P*-value = 0.64). Additionally, more than one withdrawal symptom was reported by 55.1% of patients in the ergotamine group, significantly higher than in the triptan group (6.8%, *P*-value < 0.001), but similar to the dual group (50.0%, *P*-value = 0.80).

**Table 2:** Withdrawal symptoms among patients with ergotamine-MOH, triptan-MOH and dual-MOH

Parameters	Total (*n* = 117)	Ergotamine (*n* = 61)	Triptans (*n* = 44)	Dual (*n* = 12)	*P*-value*			
					Ergotamine vs. triptans	Ergotamine vs. Dual	Triptans vs. Dual	Overall
Withdrawal symptoms – n (%)	90 (76.9)	53 (87.0)	27 (61.4)	10 (83.3)	0.02^†^	0.74	0.15	0.01^†^
Symptoms – n (%)								
Nausea/vomiting	28 (23.9)	22 (36.1)	0 (0.0)	6 (50.0)	< 0.001^†^	0.36	< 0.001^†^	< 0.001^†^
Loss of appetite	14 (12.0)	12 (19.7)	1 (2.3)	1 (8.3)	0.008^†^	0.35	0.32	0.02^†^
Agitation	17 (14.5)	5 (8.2)	9 (20.5)	3 (25.0)	0.07	0.09	0.73	0.12
Sleep disturbance	31 (26.5)	18 (29.5)	9 (20.5)	4 (33.3)	0.30	0.79	0.35	0.50
Rebound headache	52 (44.4)	35 (57.4)	11 (25.0)	6 (50.0)	0.001^†^	0.64	0.10	0.004^†^
> 1 symptom	42 (35.9)	33 (55.1)	3 (6.8)	6 (50.0)	< 0.001^†^	0.80	< 0.001^†^	< 0.001^†^

*Abbreviations: MOH* Medication-overuse headache

*Pairwise comparison values were Bonferroni-adjusted, with a significance threshold of *P* < 0.02. For overall comparisons, a *P*-value < 0.05 was considered statistically significant

^†^Statistically significant

### Factors associated with withdrawal symptoms

Table 3 demonstrated the multivariate logistic regression analysis for withdrawal symptoms. Female patients had 4.5-fold increased crude odds of experiencing withdrawal symptoms compared to males (OR = 4.5, 95% CI 1.7–11.9, *P*-value = 0.003), and this association remained significant after adjusting for potential confounders (adjusted OR (aOR) 4.3, 95% CI 1.6–12.0, *P*-value = 0.005). Similarly, ergotamine use was significantly associated with an increased likelihood of experiencing withdrawal symptoms (aOR = 3.2, 95% CI 1.2–9.0, *P*-value = 0.02).

**Table 3:** Factors associated with withdrawal symptoms of migraine-specific abortive treatment

Parameters	Crude OR (95% CI)	*P*-value	Adjusted OR^a^ (95% CI)	*P*-value
Female	4.5 (1.7–11.9)	0.003	4.3 (1.6–12.0)	0.005
Ergotamine	3.4 (1.3–8.6)	0.01	3.2 (1.2–9.0.2.0)	0.02

*Abbreviations: CI* Confidence interval, *HIT-6* Headache Impact Test-6, *NSAIDs* Non-steroidal anti-inflammatory drugs, *OR* Odds ratio

^a^Adjusted for age, acetaminophen, NSAIDs, opioid, HIT-6 score, baseline monthly headache days, and prior preventive medication used

### Treatment of MOH

As shown in Table 4, patients with ergotamine-MOH were significantly more likely to require inpatient treatment compared to those with triptan-MOH (39.3% vs. 15.9%, *P*-value = 0.01), while the dual-MOH group had the highest hospitalization rate (75.0%). All patients received corticosteroid treatment in accordance with the institutional detoxification protocol. Regarding preventive strategies, similar classes of preventive medications were prescribed across all groups. Topiramate was the most commonly used agent, prescribed in over half of patients in each group (57.4% in ergotamine-MOH, 52.3% in triptan-MOH, and 58.3% in dual-MOH). Tricyclic antidepressants (TCAs) and selective serotonin or serotonin-norepinephrine reuptake inhibitors (SSRIs/SNRIs) were the next most frequently used. The majority of patients in all groups received combination preventive therapy, with more than 90% receiving at least two preventive medications (93.4%, 88.6%, and 100.0%, respectively).

**Table 4:** MOH treatment among patients with ergotamine-MOH, triptan-MOH and dual-MOH

Parameters	Total (*n* = 117)	Ergotamine (*n* = 61)	Triptans (*n* = 44)	Dual (*n* = 12)	*P*-value*			
					Ergotamine vs. triptans	Ergotamine vs. Dual	Triptans vs. Dual	Overall
IPD treatment – n (%)	40 (34.2)	24 (39.3)	7 (15.9)	9 (75.0)	0.01^†^	0.03^†^	< 0.001^†^	< 0.001^†^
Steroid – n (%)	117 (100.0)	61 (100.0)	44 (100.0)	12 (100.0)	1.00	1.00	1.00	1.00
Preventive medication – n (%)								
CGRP antagonist	25 (21.4)	17 (27.9)	6 (13.6)	2 (16.7)	0.10	0.72	1.00	0.20
Topiramate	65 (55.6)	35 (57.4)	23 (52.3)	7 (58.3)	0.69	1.00	0.76	0.86
Valproate	44 (37.6)	22 (36.1)	18 (40.9)	4 (33.3)	0.69	1.00	0.75	0.84
Beta-blockers	13 (11.1)	6 (9.8)	7 (15.9)	0 (0.0)	0.38	0.58	0.33	0.27
Flunarizine	14 (12.0)	6 (9.8)	6 (13.6)	2 (16.7)	0.55	0.61	1.00	0.73
TCAs	58 (49.6)	28 (45.9)	22 (50.0)	8 (66.7)	0.70	0.22	0.35	0.42
SSRI/SNRIs	53 (43.3)	30 (49.2)	20 (45.5)	3 (25.0)	0.84	0.20	0.32	0.31
Botulinum toxin injection	6 (5.1)	4 (6.6)	2 (4.6)	0 (0.0)	1.00	1.00	1.00	0.63
> 1 preventive medication	108 (92.3)	57 (93.4)	39 (88.6)	12 (100.0)	0.39	0.36	0.22	0.38

*Abbreviations: CGRP* Calcitonin gene-related peptide, *IPD* Inpatient department, *MOH* Medication-overuse headache, *SNRI* Serotonin-norepinephrine reuptake inhibitor, *SSRI* Selective serotonin reuptake inhibitor, *TCA* Tricyclic antidepressant

*Pairwise comparison *P*-values were Bonferroni-adjusted, with a significance threshold of *P* < 0.02. For overall comparisons, a *P*-value < 0.05 was considered statistically significant

^†^Statistically significant

### Clinical outcomes

**Fig. 2:**
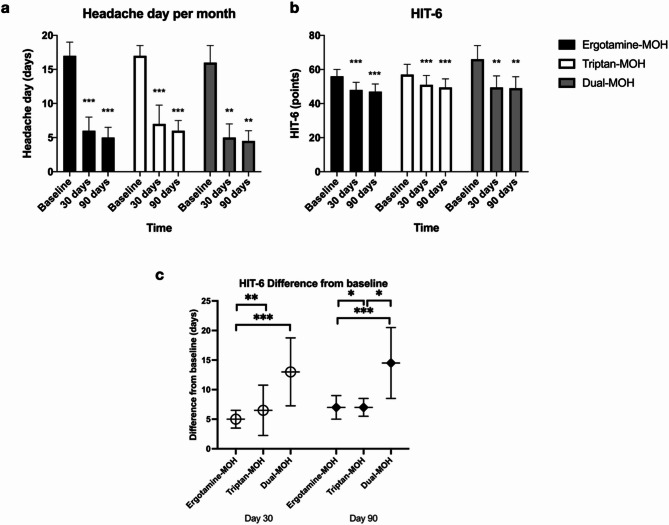
Clinical outcome. **a** Headache day per month at 30 days and 90 days compared to baseline. **b** HIT-6 at 30 days and 90 days compared to baseline. **c** HIT-6 score difference at 30 days and 90 days compared to baseline. HIT-6, Headache Impact Test-6, MOH, medication-overuse headache. Pairwise comparison *P*-values were Bonferroni-adjusted, with a significance threshold of *P* < 0.02. *P*-value: * < 0.05, ** <0.01, *** < 0.001

**Fig. 3:**
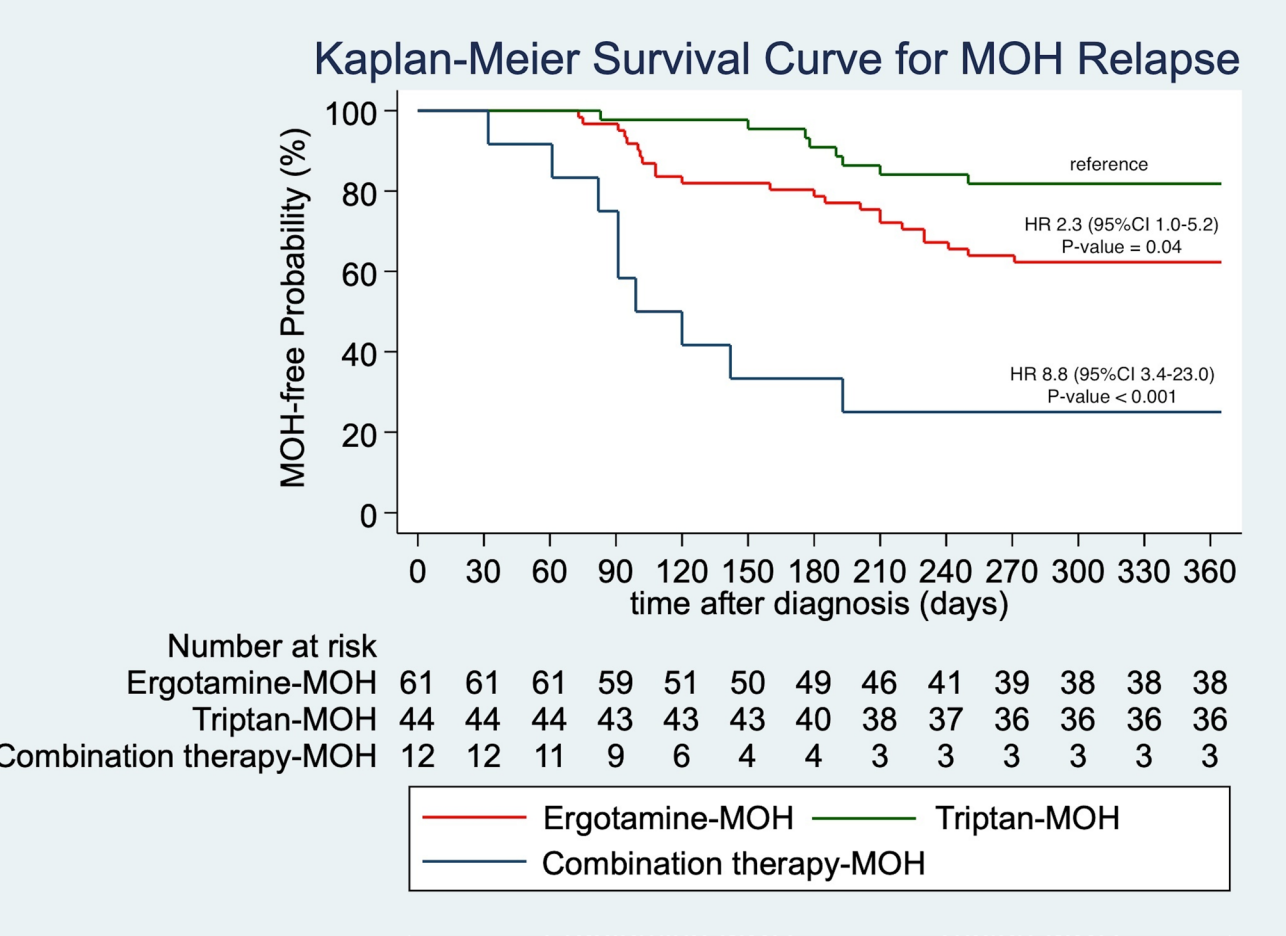
Time to First MOH Relapse. Kaplan-Meier survival curve illustrating the time to first MOH relapse across all groups over a 12-month follow-up period. Compared to triptan-MOH, ergotamine-MOH and dual-MOH carry a higher risk of relapse, with hazard ratios of 2.3 (95% CI, 1.0–5.2) and 8.8 (95% CI, 3.4–23.0), respectively. CI, confidence interval; MOH, medication-overuse headache

All three MOH groups demonstrated significant clinical improvements from baseline in both HIT-6 scores and monthly headache days at 30 and 90 days (Fig. 2a and b). However, the magnitude of improvement varied across groups. Patients with ergotamine-MOH exhibited the smallest reduction in HIT-6 scores, while those with dual-MOH experienced the most substantial improvement. At 30 days, the median HIT-6 reduction was significantly smaller in the ergotamine-MOH group compared to both the triptan-MOH (5.0 vs. 6.5 points, *P*-value < 0.001) and dual-MOH groups (5.0 vs. 13.0 points, *P*-value < 0.001). This pattern remained at 90 days, with the dual-MOH group showing significantly greater improvement than either ergotamine-MOH (14.5 vs. 7.0 points, *P*-value < 0.001) or triptan-MOH (14.5 vs. 7.0 points, *P*-value = 0.01). The difference between ergotamine-MOH and triptan-MOH at 90 days approached the adjusted significance threshold (*P*-value = 0.01) (Fig. 2c). Despite these differences in symptom severity, the absolute number of headache days per month and the reduction from baseline were comparable across groups, with no statistically significant pairwise differences at either time point. Subjective satisfaction scores were similarly high across all groups, with a median of 8.0 out of 10 (IQR 8.0–9.0). MOH recurrence was observed in 34.2% of the total cohort within 12 months, with the highest rate in the dual-MOH group (75.0%), followed by ergotamine-MOH (37.7%) and triptan-MOH (18.2%). The dual-MOH group had a significantly higher recurrence risk than either the ergotamine-MOH (*P*-value < 0.001) or triptan-MOH group (*P*-value < 0.001). Additionally, time to MOH recurrence was significantly shorter in the dual-MOH group (median 91.0 days) compared to both the ergotamine-MOH (160.0 days, *P*-value < 0.001) and triptan-MOH groups (184.0 days, *P*-value < 0.001), indicating a more refractory course. However, after six months, the MOH recurrence rates were comparable among the three groups, with no statistically significant differences observed (Overall *P*-values = 0.61). See Supplementary Tables (Table S1-S4) and Fig. 3.

## Discussion

Our study demonstrated that withdrawal symptoms related to migraine-specific abortive treatments were surprisingly common, with at least one-third of patients reporting more than one symptom. Ergotamine overuse appeared to result in a greater number of withdrawal symptoms. Further analysis identified female gender and ergotamine use as risk factors for withdrawal symptoms. The relapse rate of MOH varied significantly based on the type of migraine-specific abortive medication initially overused. These findings suggest that ergotamine overuse not only increases withdrawal symptom severity but also predisposes patients to a higher likelihood of relapse. This trend is even more pronounced in dual-MOH patients, who exhibited the shortest median relapse time (91 days), emphasizing the need for more tailored treatment approaches.

The greater severity observed in the dual-MOH group compared to single-agent groups likely reflects the substantial contribution of ergotamine, given its potent receptor affinity and prolonged pharmacological effects [20]. Combined exposure to both ergotamine and triptans may synergistically heighten receptor sensitivity or dysregulation, thereby exacerbating withdrawal symptoms and increasing the risk of relapse [21]. This combination may also reinforce medication dependence through compounded receptor engagement, leading to intensified neurochemical imbalances and reinforcing dependence. Patients with ergotamine-induced MOH experienced significantly more frequent and severe withdrawal symptoms, including nausea, vomiting, loss of appetite, and rebound headaches, underscoring the substantial clinical challenges associated with ergotamine withdrawal [22]. These findings are consistent with existing literature describing the difficulty of ergotamine withdrawal due to its broad receptor activity, vasoconstrictive potency, and frequent co-formulation with caffeine, which can intensify withdrawal through additional dependence and side effects [21, 22]. Taken together, these mechanistic differences highlight why ergotamine-related MOH is often more refractory, requiring tailored withdrawal strategies, prolonged monitoring, and potentially more intensive detoxification protocols to achieve sustained remission [23].

We identified that patients with dual-MOH (overusing both ergotamine and triptans) exhibited the highest relapse rate (75%) and the shortest median time to MOH recurrence (91 days). Our findings also highlight significant differences in treatment outcomes, as reflected in improvements in HIT-6 scores across medication groups. Patients overusing ergotamine consistently exhibited less improvement at both 30 and 90 days compared to those overusing triptans or dual therapy. Notably, the magnitude of HIT-6 reduction in the triptan and dual groups exceeded the minimally important difference reported in previous studies, indicating that these improvements were not only statistically significant but also clinically meaningful in terms of reducing disability and improving quality of life. By contrast, the smaller gains observed in the ergotamine group suggest a more refractory clinical course. This discrepancy likely reflects underlying pathophysiological differences among MOH subtypes and highlights the need for more tailored withdrawal and management strategies [22]. In particular, the standardized institutional seven-day detoxification protocol currently in use may be insufficient for all cases of MOH, especially those involving ergotamine or combination therapies [6, 11, 15]. More intensive or prolonged detoxification protocols, together with closer follow-up, may therefore be necessary to optimize clinical outcomes.

Importantly, our findings highlight persistent regional deviations from international prescribing practices, notably within Thailand, where ergotamine remains widely available as an inexpensive, over-the-counter medication [11, 13]. This ease of access significantly contributes to its prevalent misuse and elevated incidence of MOH locally, underscoring the need for targeted education and policy adjustments to shift prescribing habits toward safer alternatives, such as triptans [6]. A similar pattern has been observed in Latin America, where ergotamine is also widely sold over the counter at low prices. Data from the COMOESTAS and COMOESTAS-2 studies confirmed that ergotamine overuse remains highly prevalent in this region, reflecting availability and limited access to triptans [5, 24–26]. In this context, regulation of over-the-counter ergotamine sales, combined with broader access to affordable triptans, may represent an effective strategy to reduce misuse and recurrence risk. Beyond regulatory changes, patient and provider education campaigns are also essential to raise awareness of the risks associated with ergotamine overuse and to encourage early intervention. Our analysis also identified demographic predictors of withdrawal symptoms, notably a higher risk among female patients. The increased susceptibility in women may be related to hormonal influences, differences in pain processing, or variations in medication metabolism, highlighting the importance of personalized clinical management strategies for this subgroup [27–29].

Given the complexity of real-world clinical management, the interpretation of our findings may have been influenced by the concomitant use of abortive and preventive medications. A high proportion of patients in all groups reported frequent use of additional abortive agents—particularly acetaminophen and NSAIDs—which may independently contribute to withdrawal symptoms and complicate attribution of outcomes solely to ergotamine or triptan overuse. To mitigate this potential confounding, we conducted multivariable logistic regression analyses adjusting for concomitant medication use, HIT-6 scores, and headache frequency at baseline. Even after adjustment, ergotamine use remained significantly associated with more severe withdrawal symptoms and higher relapse rates, supporting the robustness of our findings. Nonetheless, we recognize the complexity of polypharmacy in real-world MOH management and the inherent limitations of retrospective observational data.

The institutional standardized treatment approach across all medication groups, primarily consisting of steroid-based regimens complemented by preventive medications (e.g., topiramate, tricyclic antidepressants, and Calcitonin gene-related peptide (CGRP) targeted therapies), underscores the substantial influence of specific overused medications on clinical outcomes despite uniform preventive strategies [30]. Although CGRP targeted therapies are recommended for MOH prevention in both international and national guidelines, their use remains limited in many countries due to cost, accessibility, and lack of reimbursement. Unlike CGRP-targeted therapies, which can often be initiated without prior detoxification, the oral preventive medications commonly used in our setting—such as antiepileptics and antidepressants—require a structured withdrawal regimen. In our study, corticosteroid bridging remains a mainstay, particularly in ergotamine overuse where withdrawal is more severe. While the effectiveness of steroid regimens in MOH remains controversial, most existing studies have focused on patients overusing triptans. Therefore, these findings may not be fully applicable to ergotamine-induced MOH, underscoring the need for targeted strategies in this subgroup [4, 19]. This limitation may contribute to a persistently high recurrence rate of MOH, as access to more effective treatments remains restricted. Despite conflicting evidence regarding its efficacy in MOH withdrawal, steroids continue to be incorporated as adjunctive therapy in detoxification protocols [9, 31]. This real-world observation provides critical insights for clinicians in tailoring therapeutic decisions to mitigate relapse risks and highlights the broader implications of public policy on MOH treatment accessibility and reimbursement.

In comparison with international literature, our findings confirm the greater burden of withdrawal symptoms associated with ergotamine [22]. However, they also highlight distinctive local characteristics, particularly the prevalent use of ergotamine, contrasting sharply with global prescribing trends that favor triptans [11]. These discrepancies may reflect local prescribing practices, medication availability, and patient preferences, reinforcing the necessity of contextualizing clinical guidelines and recommendations within specific healthcare environments to enhance MOH management effectively.

This study has several strengths. Primarily, this is one of the first real-world, registry-based comparative studies from Southeast Asia to examine ergotamine-MOH and triptan-MOH in a population where ergotamine remains widely used. Most previous research comes from Western countries where triptans dominate migraine management and ergotamine use has sharply declined. Our findings highlight that, despite global prescribing trends, ergotamine continues to pose a substantial MOH burden in resource-limited settings due to its affordability and over-the-counter availability. Additionally, our detailed analysis provides meaningful contributions to understanding the comparative effectiveness of migraine-specific abortive medications, which can aid in clinical decision-making for managing MOH. However, as this is a retrospective study, some data may be missing. The relatively small sample size, especially in subgroup comparisons, may limit the statistical power to detect subtle differences between treatment groups. Furthermore, institutional and national clinical guidelines in Thailand may differ from those in other institutions or countries due to variations in healthcare contexts and national policies, which can potentially influence treatment protocols and recommendations. Lastly, the ergotamine formulation available in our country is a fixed-dose combination with caffeine. While caffeine may have synergistic or confounding effects on withdrawal symptoms and treatment response, this reflects the actual clinical formulation used in practice, reinforcing the real-world applicability of our findings. However, it should be noted that in regions where ergotamine-only formulations are prescribed or where caffeine-containing combinations are uncommon, the magnitude and profile of withdrawal symptoms or relapse risk may differ. This distinction should be considered when interpreting the global generalizability of our results. Further studies are required to evaluate the long-term outcomes of MOH treatment and to develop personalized detoxification protocols tailored to individual patient characteristics, particularly for those with severe withdrawal symptoms and high relapse risk.

## Conclusion

Ergotamine-associated MOH presents with more severe withdrawal symptoms and slower recovery compared with triptan-induced MOH. The high recurrence rate, particularly in dual-MOH patients, highlights the need for tailored detoxification protocols. Limited access to CGRP targeted therapies due to reimbursement policies may further hinder optimal treatment outcomes. Individualized management strategies, along with healthcare policy changes to improve access to evidence-based therapies, are crucial. Future research should explore alternative withdrawal strategies and advocate for broader access to evidence-based preventive therapies.

## Supplementary Information

Supplementary Material 1.

## Data Availability

The study data are available from the corresponding author upon reasonable request, provided that permission has been obtained from all contributing authors.

## Abbreviations

5-HT: 5-Hydroxytryptamine
CGRP: Calcitonin gene-related peptide
CI: Confidence interval
ICHD-3: International Classification of Headache Disorders, 3rd edition
IQR: Interquartile range
HR: Hazard ratio
HIT-6: Headache Impact Test-6
MOH: Medication overused headache
NSAIDs: Non-steroidal anti-inflammatory drugs
OR: Odds ratio
SD: Standard deviation
SNRI: Serotonin-norepinephrine reuptake inhibitor
SSRI: Selective serotonin reuptake inhibitor
TCA: Tricyclic antidepressant
VAS: Visual analog scale
