# Supplementary material for: Ergotamine and triptans induced medication-overuse headache: a real-world population-based comparative study from the Northern Thai headache registry
Source: J Headache Pain. 2025 Oct 2;26(1):198. doi: 10.1186/s10194-025-02161-6 (PMC12492508; doi:10.1186/s10194-025-02161-6)
Supplement: Supplementary file 1 — Supplementary Material 1. [file 10194_2025_2161_MOESM1_ESM.docx]

Table S1 Clinical outcomes among patients with ergotamine-MOH, triptan-MOH and dual-MOH

| **Parameters** | **Total**  **(n=117)** | **Ergotamine**  **(n=61)** | **Triptans**  **(n=44)** | **Dual (n=12)** | **P-value*** | | | |
| --- | --- | --- | --- | --- | --- | --- | --- | --- |
|  |  |  |  |  | Ergotamine vs triptans | Ergotamine vs Dual | Triptans vs Dual | Overall |
| HIT-6, points – median (IQR) | | | | | | | | |
| 30 days | 50.0 (43.0, 54.0) | 48.0 (43.0, 52.0) | 51.0 (43.0, 54.0) | 49.5 (41.5, 53.0) | 0.27 | 0.79 | 0.70 | 0.55 |
| 90 days | 49.0 (42.0, 52.0) | 47.0 (41.0, 50.0) | 49.5 (43.0, 53.0) | 49.0 (40.5, 53.0) | 0.12 | 0.79 | 0.70 | 0.32 |
| HIT-6 difference from baseline, points – median (IQR) | | | | | | | | |
| 30 days | 5.0 (5.0, 11.0) | 5.0 (4.0, 7.0) | 6.5 (5.0, 13.5) | 13.0 (8.5, 19.0) | 0.001^†^ | <0.001^†^ | 0.07 | <0.001^†^ |
| 90 days | 7.0 (6.0, 13.0) | 7.0 (6.0, 10.0) | 7.0 (7.0, 13.0) | 14.5 (9.0, 21.0) | 0.02^†^ | <0.001^†^ | 0.02^†^ | 0.002^†^ |
| Headache days per months, days – median (IQR) | | | | | | | | |
| 30 days | 6.0 (5.0, 8.0) | 6.0 (5.0, 9.0) | 7.0 (4.5, 8.0) | 5.0 (4.0, 8.0) | 0.89 | 0.40 | 0.37 | 0.66 |
| 90 days | 6.0 (4.0, 7.0) | 5.0 (4.0, 7.0) | 6.0 (4.0, 7.0) | 4.5 (3.0, 6.0) | 0.55 | 0.19 | 0.09 | 0.25 |
| Headache days per months difference from baseline, days – median (IQR) | | | | | | | | |
| 30 days | 11.0 (9.0, 13.0) | 11.0 (8.0, 13.0) | 10.5 (9.0, 12.0) | 11.5 (11.0, 12.5) | 0.55 | 0.56 | 0.22 | 0.54 |
| 90 days | 12.0 (10.0, 14.0) | 12.0 (10.0, 14.0) | 11.5 (9.0, 13.0) | 12.5 (11.5, 14.0) | 0.37 | 0.31 | 0.10 | 0.26 |
| Satisfaction, points – median (IQR) | 8.0 (8.0, 9.0) | 8.0 (8.0, 9.0) | 8.0 (8.0, 9.0) | 8.0 (8.0, 9.0) | 1.00 | 1.00 | 1.00 | 0.98 |
| MOH recurrence | | | | | | | | |
| MOH recurrence within 12 months– n (%) | 40 (34.2) | 23 (37.7) | 8 (18.2) | 9 (75.0) | 0.01^†^ | 0.01^†^ | <0.001^†^ | 0.001^†^ |
| Time to MOH recurrence, days – median (IQR) | 146.0 (94.5, 205.5) | 160.0 (100.0, 220.0) | 184.0 (163.0, 201.5) | 91.0 (82.0, 120.0) | 0.02^†^ | <0.001^†^ | <0.001^†^ | <0.001^†^ |

* Pairwise comparison *P*-values were Bonferroni-adjusted, with a significance threshold of *P* < 0.02. For overall comparisons, a *P*-value < 0.05 was considered statistically significant.

^†^ Statistically significant

**Abbreviations:** HIT-6; Headache Impact Test-6, IQR; interquartile range, MOH; medication overused headache.

Table S2 HIT-6 improvement stratified by follow-up duration compared to baseline

| **Group – median (IQR)** | **HIT-6 (points)** | **P-value** | **HIT-6 difference (points)** |
| --- | --- | --- | --- |
| **Ergotamine-MOH (n=61)** | | | |
| Baseline | 56.0 (50.0, 58.0) | (reference) | - |
| 30 days | 48.0 (43.0, 52.0) | <0.001 | 5.0 (4.0, 7.0) |
| 90 days | 47.0 (41.0, 50.0) | <0.001 | 7.0 (6.0, 10.0) |
| **Triptan-MOH (n=44)** | | | |
| Baseline | 57.0 (55.0, 67.0) | (reference) | - |
| 30 days | 51.0 (43.0, 54.0) | <0.001 | 6.5 (5.0, 13.5) |
| 90 days | 49.5 (43.0, 53.0) | <0.001 | 7.0 (7.0, 13.0) |
| **Dual-MOH (n=12)** | | | |
| Baseline | 66.0 (54.0, 70.0) | (reference) | - |
| 30 days | 49.5 (41.5, 53.0) | 0.002 | 13.0 (8.5, 19.0) |
| 90 days | 49.0 (40.5, 53.0) | 0.002 | 14.5 (9.0, 21.0) |

**Abbreviations:** HIT-6; Headache Impact Test-6, IQR; interquartile range, MOH; medication overused headache.

Table S3 Clinical improvement stratified by follow-up duration compared to baseline

| **Group – median (IQR)** | **Headache day per month (days)** | **P-value** | **Headache day per month difference (days)** |
| --- | --- | --- | --- |
| **Ergotamine-MOH (n=61)** | | | |
| Baseline | 17.0 (15.0, 19.0) | (reference) | - |
| 30 days | 6.0 (5.0, 9.0) | <0.001 | 11.0 (8.0, 13.0) |
| 90 days | 5.0 (4.0, 7.0) | <0.001 | 12.0 (10.0, 14.0) |
| **Triptan-MOH (n=44)** | | | |
| Baseline | 17.0 (15.5, 18.5) | (reference) | - |
| 30 days | 7.0 (4.5, 8.0) | <0.001 | 10.5 (9.0, 12.0) |
| 90 days | 6.0 (4.0, 7.0) | <0.001 | 11.5 (9.0, 13.0) |
| **Dual-MOH (n=12)** | | | |
| Baseline | 16.0 (15.0, 20.0) | (reference) | - |
| 30 days | 5.0 (4.0, 8.0) | 0.002 | 11.5 (11.0, 12.5) |
| 90 days | 4.5 (3.0, 6.0) | 0.002 | 12.5 (11.5, 14.0) |

**Abbreviations:** IQR; interquartile range, MOH; medication overused headache.

Table S4 MOH recurrence stratified by within 3-month, 3-6 months, and more than 6 months

| **MOH recurrence – n (%)** | **Ergotamine**  **(n=61)** | **Triptans**  **(n=44)** | **Dual (n=12)** | **P-value*** | | | |
| --- | --- | --- | --- | --- | --- | --- | --- |
|  |  |  |  | Ergotamine vs triptans | Ergotamine vs Dual | Triptans vs Dual | Overall |
| Within 3 months | 2 (3.3) | 1 (2.3) | 3 (25.0) | 1.00 | 0.03 | 0.03 | 0.02^†^ |
| 3-6 months | 11 (18.0) | 3 (6.8) | 5 (41.7) | 0.15 | 0.12 | 0.008 | 0.02^†^ |
| More than 6 months | 10 (16.4) | 4 (9.1) | 1 (8.3) | 0.39 | 0.68 | 1.00 | 0.61 |

* Pairwise comparison *P*-values were Bonferroni-adjusted, with a significance threshold of *P* < 0.02. For overall comparisons, a *P*-value < 0.05 was considered statistically significant.

^†^ Statistically significant

**Abbreviations:** MOH; medication overused headache.
